# [*N*,*N*-Bis(2,6-diisopropyl­phen­yl)pent-2-ene-2,4-diiminato(1−)]bis­(1,2,4-diaza­phosphol-1-yl)aluminium(III)

**DOI:** 10.1107/S1600536810049007

**Published:** 2010-11-27

**Authors:** Dongming Yang, Chengfu Pi, Yuqiang Ding, Wenjun Zheng

**Affiliations:** a School of Chemical and Material Engineering, Jiangnan University, 1800 Lihu Road, Wuxi, Jiangsu Province 214122, People’s Republic of China; b College of Qianjiang, Hangzhou Normal University, Wenyi Road 222, Hangzhou Zhejiang Province 310012, People’s Republic of China; c Institute of Organic Chemistry, Shanxi Normal University, 1 Gongyuan Street, Linfen, Shanxi Province 041004, People’s Republic of China

## Abstract

In the title compound, [Al(C_29_H_41_N_2_)(C_2_H_2_N_2_P)_2_], the Al^III^ atom is coordinated by four N atoms from β-diketiminate and 1,2,4-diaza­phospho­lide ligands in a slightly distorted tetra­hedral fashion.

## Related literature

For similar related 1,2,4-diaza­phospho­lide complexes, see: Schmidpeter & Willhalm (1984 ▶); Cui *et al.* (2000 ▶); Ding *et al.* (2001 ▶); Kumar *et al.* (2004 ▶, 2005 ▶); Zheng *et al.* (2006 ▶); Wan *et al.* (2008 ▶); Pi *et al.* (2008 ▶, 2009 ▶).
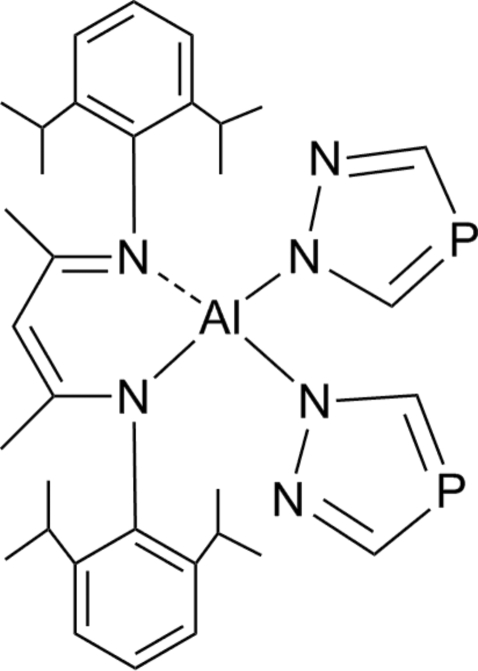


## Experimental

### 

#### Crystal data

[Al(C_29_H_41_N_2_)(C_2_H_2_N_2_P)_2_]
*M* *_r_* = 614.67
Triclinic, 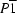

*a* = 10.578 (4) Å
*b* = 12.578 (5) Å
*c* = 13.498 (5) Å
α = 92.059 (5)°
β = 98.766 (5)°
γ = 96.516 (5)°
*V* = 1760.8 (11) Å^3^
*Z* = 2
Mo *K*α radiation
μ = 0.18 mm^−1^
*T* = 293 K
0.35 × 0.20 × 0.20 mm

#### Data collection

Bruker SMART APEXII CCD area-detector diffractometer
Absorption correction: multi-scan (*SADABS*; Bruker, 2001 ▶) *T*_min_ = 0.940, *T*_max_ = 0.965
7337 measured reflections
6082 independent reflections
4238 reflections with *I* > 2σ(*I*)
*R*_int_ = 0.037

#### Refinement

*R*[*F* ^2^ > 2σ(*F* ^2^)] = 0.075
*wR*(*F* ^2^) = 0.224
*S* = 1.02
6082 reflections
389 parameters
H-atom parameters constrained
Δρ_max_ = 0.47 e Å^−3^
Δρ_min_ = −0.56 e Å^−3^

### 

Data collection: *APEX2* (Bruker, 2005 ▶); cell refinement: *SAINT* (Bruker, 2005 ▶); data reduction: *SAINT*; program(s) used to solve structure: *SIR97* (Altomare *et al.*, 1999 ▶); program(s) used to refine structure: *SHELXL97* (Sheldrick, 2008 ▶); molecular graphics: *PLATON* (Spek, 2009 ▶); software used to prepare material for publication: *SHELXL97*.

## Supplementary Material

Crystal structure: contains datablocks global, I. DOI: 10.1107/S1600536810049007/bq2246sup1.cif

Structure factors: contains datablocks I. DOI: 10.1107/S1600536810049007/bq2246Isup2.hkl

Additional supplementary materials:  crystallographic information; 3D view; checkCIF report

## Figures and Tables

**Fig. 1.**
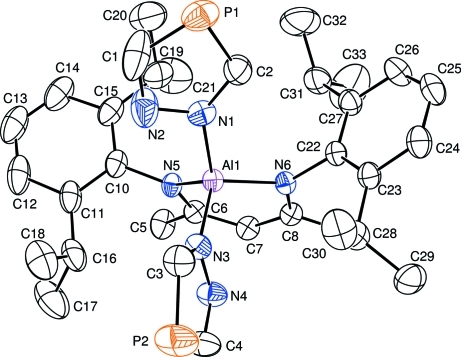
The structure of the title complex with the atom numbering scheme. The thermal displacements are drawn at the 30% probability level. Hydrogen atoms are omitted for clarity.

## supplementary crystallographic information

### Comment

Recently, the investigation of 1,2,4-diazaphospholide complexes has attracted considerable interest (Zheng *et al.*, 2006-2009). On the other hand, aluminum hydride complexes with bulky beta-diketiminato ligand [HC(CMeNAr)2] AlH2 have been evidenced to be a reactive species (Roesky *et al.*, 2000-2005). Herein, we report a centrosymmetric complex which was synthesized by the reaction of [HC(CMeNAr)2] AlH2 with 1*H*-1,2,4-diazaphosphole in hexane at room temperature. As illustrated in Fig. 1, the Al^III^ ion was coordinated by four nitrogen atoms of 2,6-iPr2C6H3NC(Me)C(H)C(Me)N and 1,2,4-diazaphospholide ligands. The two nitrogen atoms from the 2,6-iPr2C6H3NC(Me)C(H)C(Me)NH ligand form a six-member ring with the aluminum center, and the other two nitrogen atoms from the 1,2,4-diazaphospholide ligands coordinate to aluminum atom in a eta(1) mode. The four nitrogen atoms are arranged in a slightly distorted tetrahedral fashion. The plane of the six-membered ring C3—N2—Al is nearly perpendicular to the 1,2,4-diazaphospholide heterocycle rings.

### Experimental

All manipulations were carried out under an argon atmosphere using standard Schlenk techniques. Hexane was dried over sodium and freshly distilled prior to use. 0.481 g [2,6-iPr2C6H3NC(Me)C(H)C(Me)N]AlH2 (1 eq.) and 0.172 g (2 eq.) 1,2,4-Dia-zaphosphole were dissolved in 20 ml toulent. The mixture was stirred for 24 h at room temperature and the solvent was then removed and dried *in vacuo*. The residua was extracted with 15 ml hexane and the solution was concentrated to about 5 ml to afford colorless crystals at -30°C for several days (yield: 0.32 g. 50%).

### Refinement

All H atoms were placed in geometrically idealized positions and constrained to ride on their parent atoms with C—H distances of 0.93–0.96 Å, and *U*_iso_(H) = 1.2–1.5 times of those of their parent atoms.

### Crystal data

**Table:**

[Al(C_29_H_41_N_2_)(C_2_H_2_N_2_P)_2_]	*Z* = 2
*M**_r_* = 614.67	*F*(000) = 656
Triclinic, *P*1	*D*_x_ = 1.159 Mg m^−^^3^
Hall symbol: -P 1	Mo *K*α radiation, λ = 0.71073 Å
*a* = 10.578 (4) Å	Cell parameters from 872 reflections
*b* = 12.578 (5) Å	θ = 3.4–25.6°
*c* = 13.498 (5) Å	µ = 0.18 mm^−^^1^
α = 92.059 (5)°	*T* = 293 K
β = 98.766 (5)°	Sheet, yellow
γ = 96.516 (5)°	0.35 × 0.20 × 0.20 mm
*V* = 1760.8 (11) Å^3^	

### Data collection

**Table:**

Bruker SMART APEXII CCD area-detector diffractometer	6082 independent reflections
Radiation source: fine-focus sealed tube	4238 reflections with *I* > 2σ(*I*)
graphite	*R*_int_ = 0.037
phi and ω scans	θ_max_ = 25.0°, θ_min_ = 1.6°
Absorption correction: multi-scan (*SADABS*; Bruker, 2001)	*h* = −12→10
*T*_min_ = 0.940, *T*_max_ = 0.965	*k* = −11→14
7337 measured reflections	*l* = −16→12

### Refinement

**Table:**

Refinement on *F*^2^	0 restraints
Least-squares matrix: full	H-atom parameters constrained
*R*[*F*^2^ > 2σ(*F*^2^)] = 0.075	*w* = 1/[σ^2^(*F*_o_^2^) + (0.1531*P*)^2^ + ] where *P* = (*F*_o_^2^ + 2*F*_c_^2^)/3
*wR*(*F*^2^) = 0.224	(Δ/σ)_max_ < 0.001
*S* = 1.01	Δρ_max_ = 0.47 e Å^−^^3^
6082 reflections	Δρ_min_ = −0.56 e Å^−^^3^
389 parameters	

### Special details

**Table:**

Geometry. All e.s.d.'s (except the e.s.d. in the dihedral angle between two l.s. planes) are estimated using the full covariance matrix. The cell e.s.d.'s are taken into account individually in the estimation of e.s.d.'s in distances, angles and torsion angles; correlations between e.s.d.'s in cell parameters are only used when they are defined by crystal symmetry. An approximate (isotropic) treatment of cell e.s.d.'s is used for estimating e.s.d.'s involving l.s. planes.
Refinement. Refinement of *F*^2^ against ALL reflections. The weighted *R*-factor *wR* and goodness of fit *S* are based on *F*^2^, conventional *R*-factors *R* are based on *F*, with *F* set to zero for negative *F*^2^. The threshold expression of *F*^2^ > σ(*F*^2^) is used only for calculating *R*-factors(gt) *etc*. and is not relevant to the choice of reflections for refinement. *R*-factors based on *F*^2^ are statistically about twice as large as those based on *F*, and *R*- factors based on ALL data will be even larger.

### Fractional atomic coordinates and isotropic or equivalent isotropic displacement parameters (Å^2^)

**Table:**

	*x*	*y*	*z*	*U*_iso_*/*U*_eq_	
Al1	0.22665 (7)	0.79946 (6)	0.78731 (6)	0.0419 (3)	
P1	−0.16571 (10)	0.71060 (10)	0.62146 (10)	0.0880 (4)	
P2	0.18913 (13)	1.08771 (11)	0.97295 (11)	0.1047 (5)	
N1	0.0608 (2)	0.7524 (2)	0.7248 (2)	0.0574 (6)	
N2	−0.0133 (3)	0.7202 (4)	0.7910 (3)	0.1033 (13)	
N3	0.2190 (2)	0.9204 (2)	0.86843 (18)	0.0537 (6)	
N4	0.3412 (2)	0.9627 (2)	0.9076 (2)	0.0652 (7)	
N5	0.3149 (2)	0.70050 (18)	0.86114 (16)	0.0479 (6)	
N6	0.3367 (2)	0.83042 (17)	0.69477 (16)	0.0435 (5)	
C1	−0.1375 (5)	0.6917 (5)	0.7425 (4)	0.125 (2)	
H1	−0.2025	0.6632	0.7766	0.149*	
C2	−0.0057 (4)	0.7533 (3)	0.6309 (3)	0.0762 (10)	
H2	0.0327	0.7753	0.5763	0.091*	
C3	0.1292 (3)	0.9771 (3)	0.8954 (3)	0.0717 (9)	
H3	0.0415	0.9581	0.8738	0.086*	
C4	0.3387 (4)	1.0485 (3)	0.9637 (3)	0.0806 (11)	
H4	0.4141	1.0871	0.9975	0.097*	
C5	0.5225 (3)	0.6473 (3)	0.9408 (3)	0.0738 (10)	
H5A	0.5084	0.6655	1.0076	0.111*	
H5B	0.6123	0.6638	0.9364	0.111*	
H5C	0.4967	0.5722	0.9253	0.111*	
C6	0.4445 (3)	0.7107 (2)	0.8674 (2)	0.0504 (7)	
C7	0.5112 (3)	0.7783 (2)	0.8092 (2)	0.0525 (7)	
H7	0.6000	0.7900	0.8291	0.063*	
C8	0.4642 (3)	0.8307 (2)	0.7260 (2)	0.0493 (7)	
C9	0.5580 (3)	0.8893 (3)	0.6683 (3)	0.0737 (10)	
H9A	0.5349	0.8682	0.5983	0.111*	
H9B	0.6432	0.8721	0.6920	0.111*	
H9C	0.5562	0.9651	0.6778	0.111*	
C10	0.2540 (3)	0.6173 (3)	0.9171 (2)	0.0582 (8)	
C11	0.2390 (3)	0.6407 (3)	1.0157 (3)	0.0725 (10)	
C12	0.1862 (4)	0.5554 (5)	1.0671 (4)	0.0997 (16)	
H12	0.1770	0.5676	1.1339	0.120*	
C13	0.1485 (5)	0.4568 (5)	1.0230 (5)	0.1080 (17)	
H13	0.1132	0.4027	1.0593	0.130*	
C14	0.1618 (4)	0.4355 (4)	0.9258 (4)	0.0977 (14)	
H14	0.1370	0.3667	0.8966	0.117*	
C15	0.2122 (3)	0.5161 (3)	0.8695 (3)	0.0715 (10)	
C16	0.2732 (4)	0.7513 (4)	1.0672 (3)	0.0849 (12)	
H16	0.3084	0.7988	1.0194	0.102*	
C17	0.3773 (6)	0.7541 (6)	1.1615 (3)	0.138 (2)	
H17A	0.3416	0.7163	1.2133	0.207*	
H17B	0.4057	0.8272	1.1849	0.207*	
H17C	0.4491	0.7207	1.1450	0.207*	
C18	0.1544 (5)	0.7959 (5)	1.0947 (4)	0.1133 (16)	
H18A	0.0886	0.7905	1.0366	0.170*	
H18B	0.1766	0.8697	1.1178	0.170*	
H18C	0.1231	0.7555	1.1468	0.170*	
C19	0.2203 (4)	0.4905 (3)	0.7606 (3)	0.0836 (11)	
H19	0.2347	0.5587	0.7289	0.100*	
C20	0.0958 (6)	0.4301 (5)	0.7050 (5)	0.142 (2)	
H20A	0.0760	0.3648	0.7371	0.213*	
H20B	0.1056	0.4137	0.6368	0.213*	
H20C	0.0271	0.4737	0.7059	0.213*	
C21	0.3319 (6)	0.4291 (5)	0.7488 (5)	0.149 (2)	
H21A	0.4112	0.4722	0.7761	0.224*	
H21B	0.3313	0.4118	0.6789	0.224*	
H21C	0.3242	0.3642	0.7839	0.224*	
C22	0.2938 (3)	0.8573 (2)	0.5916 (2)	0.0476 (6)	
C23	0.2616 (3)	0.9594 (2)	0.5707 (2)	0.0548 (7)	
C24	0.2151 (4)	0.9782 (3)	0.4717 (3)	0.0702 (9)	
H24	0.1928	1.0458	0.4561	0.084*	
C25	0.2012 (4)	0.8998 (4)	0.3964 (3)	0.0782 (10)	
H25	0.1699	0.9140	0.3306	0.094*	
C26	0.2336 (4)	0.8011 (3)	0.4187 (3)	0.0776 (10)	
H26	0.2243	0.7484	0.3672	0.093*	
C27	0.2802 (3)	0.7761 (3)	0.5160 (2)	0.0613 (8)	
C28	0.2762 (4)	1.0491 (3)	0.6497 (3)	0.0691 (9)	
H28	0.3124	1.0225	0.7140	0.083*	
C29	0.3710 (4)	1.1436 (3)	0.6248 (3)	0.0898 (12)	
H29A	0.4510	1.1183	0.6161	0.135*	
H29B	0.3860	1.1975	0.6789	0.135*	
H29C	0.3348	1.1737	0.5641	0.135*	
C30	0.1480 (5)	1.0861 (4)	0.6605 (3)	0.0971 (14)	
H30A	0.1175	1.1232	0.6023	0.146*	
H30B	0.1579	1.1336	0.7193	0.146*	
H30C	0.0869	1.0252	0.6669	0.146*	
C31	0.3132 (4)	0.6642 (3)	0.5355 (3)	0.0788 (10)	
H31	0.3299	0.6582	0.6084	0.095*	
C32	0.2037 (7)	0.5800 (4)	0.4934 (5)	0.143 (2)	
H32A	0.1274	0.5943	0.5193	0.214*	
H32B	0.2253	0.5107	0.5125	0.214*	
H32C	0.1885	0.5813	0.4215	0.214*	
C33	0.4358 (7)	0.6444 (5)	0.4945 (6)	0.163 (3)	
H33A	0.4168	0.6331	0.4228	0.244*	
H33B	0.4681	0.5822	0.5232	0.244*	
H33C	0.4996	0.7056	0.5117	0.244*	

### Atomic displacement parameters (Å^2^)

**Table:**

	*U*^11^	*U*^22^	*U*^33^	*U*^12^	*U*^13^	*U*^23^
Al1	0.0409 (4)	0.0430 (5)	0.0436 (4)	0.0117 (3)	0.0059 (3)	0.0077 (3)
P1	0.0608 (6)	0.0820 (7)	0.1125 (9)	0.0055 (5)	−0.0134 (5)	0.0134 (6)
P2	0.0950 (8)	0.0948 (9)	0.1276 (10)	0.0281 (7)	0.0282 (7)	−0.0450 (8)
N1	0.0461 (13)	0.0545 (15)	0.0702 (16)	0.0116 (11)	0.0003 (11)	0.0041 (12)
N2	0.069 (2)	0.160 (4)	0.084 (2)	0.013 (2)	0.0102 (17)	0.052 (2)
N3	0.0525 (13)	0.0565 (15)	0.0545 (13)	0.0158 (12)	0.0100 (11)	−0.0011 (11)
N4	0.0556 (15)	0.0683 (18)	0.0710 (17)	0.0125 (13)	0.0084 (12)	−0.0148 (14)
N5	0.0490 (13)	0.0486 (13)	0.0482 (12)	0.0112 (11)	0.0077 (10)	0.0143 (10)
N6	0.0479 (12)	0.0416 (12)	0.0439 (12)	0.0139 (10)	0.0088 (9)	0.0069 (9)
C1	0.087 (3)	0.152 (5)	0.157 (5)	0.031 (3)	0.061 (3)	0.084 (4)
C2	0.071 (2)	0.086 (3)	0.069 (2)	0.005 (2)	0.0050 (17)	0.0152 (19)
C3	0.0625 (19)	0.077 (2)	0.082 (2)	0.0228 (18)	0.0210 (16)	−0.0035 (18)
C4	0.073 (2)	0.079 (3)	0.085 (2)	0.0090 (19)	0.0056 (18)	−0.029 (2)
C5	0.062 (2)	0.081 (2)	0.083 (2)	0.0280 (18)	0.0020 (17)	0.0361 (19)
C6	0.0485 (15)	0.0505 (16)	0.0531 (15)	0.0147 (13)	0.0034 (12)	0.0080 (13)
C7	0.0419 (14)	0.0551 (17)	0.0626 (17)	0.0149 (13)	0.0074 (12)	0.0071 (14)
C8	0.0491 (15)	0.0446 (15)	0.0570 (16)	0.0123 (13)	0.0119 (12)	0.0037 (12)
C9	0.0579 (19)	0.086 (3)	0.084 (2)	0.0121 (18)	0.0261 (17)	0.026 (2)
C10	0.0487 (16)	0.066 (2)	0.0618 (18)	0.0156 (15)	0.0032 (13)	0.0298 (15)
C11	0.0610 (19)	0.100 (3)	0.0609 (19)	0.0191 (19)	0.0094 (15)	0.0404 (19)
C12	0.080 (3)	0.142 (5)	0.087 (3)	0.028 (3)	0.023 (2)	0.069 (3)
C13	0.086 (3)	0.109 (4)	0.132 (4)	0.008 (3)	0.016 (3)	0.073 (4)
C14	0.081 (3)	0.078 (3)	0.136 (4)	0.012 (2)	0.009 (3)	0.057 (3)
C15	0.0608 (19)	0.060 (2)	0.094 (3)	0.0116 (17)	0.0055 (18)	0.0335 (19)
C16	0.083 (2)	0.124 (4)	0.0481 (18)	0.015 (2)	0.0081 (17)	0.019 (2)
C17	0.114 (4)	0.229 (7)	0.066 (3)	0.032 (4)	−0.004 (3)	0.000 (4)
C18	0.112 (4)	0.148 (5)	0.089 (3)	0.034 (3)	0.029 (3)	0.012 (3)
C19	0.098 (3)	0.0468 (19)	0.105 (3)	0.0131 (19)	0.010 (2)	0.0077 (19)
C20	0.137 (5)	0.130 (5)	0.139 (5)	−0.030 (4)	−0.008 (4)	0.003 (4)
C21	0.143 (5)	0.138 (5)	0.175 (6)	0.058 (4)	0.028 (4)	−0.031 (4)
C22	0.0482 (14)	0.0521 (16)	0.0449 (14)	0.0108 (13)	0.0095 (11)	0.0087 (12)
C23	0.0603 (17)	0.0542 (17)	0.0519 (16)	0.0130 (14)	0.0088 (13)	0.0122 (13)
C24	0.078 (2)	0.072 (2)	0.064 (2)	0.0208 (19)	0.0079 (16)	0.0246 (17)
C25	0.086 (2)	0.099 (3)	0.0493 (18)	0.018 (2)	0.0036 (16)	0.0194 (19)
C26	0.092 (3)	0.091 (3)	0.0501 (18)	0.016 (2)	0.0098 (17)	−0.0016 (18)
C27	0.072 (2)	0.0618 (19)	0.0533 (17)	0.0107 (16)	0.0168 (14)	0.0032 (14)
C28	0.094 (2)	0.0515 (18)	0.0631 (19)	0.0244 (18)	0.0038 (17)	0.0110 (15)
C29	0.108 (3)	0.060 (2)	0.095 (3)	0.006 (2)	−0.002 (2)	0.013 (2)
C30	0.122 (4)	0.087 (3)	0.097 (3)	0.052 (3)	0.033 (3)	0.012 (2)
C31	0.111 (3)	0.059 (2)	0.069 (2)	0.017 (2)	0.017 (2)	−0.0058 (16)
C32	0.202 (6)	0.065 (3)	0.139 (5)	−0.008 (4)	−0.023 (4)	−0.014 (3)
C33	0.169 (6)	0.097 (4)	0.255 (8)	0.072 (4)	0.089 (6)	0.026 (5)

### Geometric parameters (Å, °)

**Table:**

Al1—N1	1.848 (3)	C16—H16	0.9800
Al1—N6	1.855 (2)	C17—H17A	0.9600
Al1—N3	1.858 (3)	C17—H17B	0.9600
Al1—N5	1.867 (2)	C17—H17C	0.9600
P1—C1	1.646 (6)	C18—H18A	0.9600
P1—C2	1.700 (4)	C18—H18B	0.9600
P2—C3	1.711 (4)	C18—H18C	0.9600
P2—C4	1.731 (4)	C19—C21	1.506 (7)
N1—N2	1.320 (4)	C19—C20	1.518 (7)
N1—C2	1.354 (4)	C19—H19	0.9800
N2—C1	1.378 (6)	C20—H20A	0.9600
N3—C3	1.336 (4)	C20—H20B	0.9600
N3—N4	1.360 (4)	C20—H20C	0.9600
N4—C4	1.301 (4)	C21—H21A	0.9600
N5—C6	1.352 (4)	C21—H21B	0.9600
N5—C10	1.460 (4)	C21—H21C	0.9600
N6—C8	1.350 (4)	C22—C23	1.393 (4)
N6—C22	1.461 (3)	C22—C27	1.397 (4)
C1—H1	0.9300	C23—C24	1.390 (4)
C2—H2	0.9300	C23—C28	1.503 (4)
C3—H3	0.9300	C24—C25	1.370 (5)
C4—H4	0.9300	C24—H24	0.9300
C5—C6	1.502 (4)	C25—C26	1.356 (5)
C5—H5A	0.9600	C25—H25	0.9300
C5—H5B	0.9600	C26—C27	1.391 (5)
C5—H5C	0.9600	C26—H26	0.9300
C6—C7	1.384 (4)	C27—C31	1.511 (5)
C7—C8	1.379 (4)	C28—C30	1.509 (6)
C7—H7	0.9300	C28—C29	1.546 (6)
C8—C9	1.498 (4)	C28—H28	0.9800
C9—H9A	0.9600	C29—H29A	0.9600
C9—H9B	0.9600	C29—H29B	0.9600
C9—H9C	0.9600	C29—H29C	0.9600
C10—C11	1.389 (5)	C30—H30A	0.9600
C10—C15	1.398 (5)	C30—H30B	0.9600
C11—C12	1.407 (6)	C30—H30C	0.9600
C11—C16	1.513 (6)	C31—C32	1.504 (7)
C12—C13	1.347 (7)	C31—C33	1.527 (7)
C12—H12	0.9300	C31—H31	0.9800
C13—C14	1.361 (7)	C32—H32A	0.9600
C13—H13	0.9300	C32—H32B	0.9600
C14—C15	1.395 (5)	C32—H32C	0.9600
C14—H14	0.9300	C33—H33A	0.9600
C15—C19	1.511 (6)	C33—H33B	0.9600
C16—C18	1.523 (6)	C33—H33C	0.9600
C16—C17	1.548 (6)		
			
N1—Al1—N6	111.60 (12)	C16—C17—H17C	109.5
N1—Al1—N3	107.50 (11)	H17A—C17—H17C	109.5
N6—Al1—N3	110.79 (11)	H17B—C17—H17C	109.5
N1—Al1—N5	116.84 (12)	C16—C18—H18A	109.5
N6—Al1—N5	99.77 (10)	C16—C18—H18B	109.5
N3—Al1—N5	110.22 (11)	H18A—C18—H18B	109.5
C1—P1—C2	86.8 (2)	C16—C18—H18C	109.5
C3—P2—C4	85.27 (17)	H18A—C18—H18C	109.5
N2—N1—C2	112.7 (3)	H18B—C18—H18C	109.5
N2—N1—Al1	110.9 (2)	C21—C19—C15	112.2 (4)
C2—N1—Al1	135.9 (3)	C21—C19—C20	110.1 (4)
N1—N2—C1	109.3 (3)	C15—C19—C20	112.1 (4)
C3—N3—N4	113.3 (3)	C21—C19—H19	107.4
C3—N3—Al1	138.0 (2)	C15—C19—H19	107.4
N4—N3—Al1	108.65 (18)	C20—C19—H19	107.4
C4—N4—N3	109.9 (3)	C19—C20—H20A	109.5
C6—N5—C10	118.2 (2)	C19—C20—H20B	109.5
C6—N5—Al1	117.43 (19)	H20A—C20—H20B	109.5
C10—N5—Al1	124.27 (18)	C19—C20—H20C	109.5
C8—N6—C22	118.4 (2)	H20A—C20—H20C	109.5
C8—N6—Al1	117.63 (18)	H20B—C20—H20C	109.5
C22—N6—Al1	123.90 (17)	C19—C21—H21A	109.5
N2—C1—P1	116.9 (3)	C19—C21—H21B	109.5
N2—C1—H1	121.6	H21A—C21—H21B	109.5
P1—C1—H1	121.6	C19—C21—H21C	109.5
N1—C2—P1	114.1 (3)	H21A—C21—H21C	109.5
N1—C2—H2	122.9	H21B—C21—H21C	109.5
P1—C2—H2	122.9	C23—C22—C27	121.4 (3)
N3—C3—P2	114.3 (3)	C23—C22—N6	120.7 (2)
N3—C3—H3	122.8	C27—C22—N6	117.8 (3)
P2—C3—H3	122.8	C24—C23—C22	117.8 (3)
N4—C4—P2	117.2 (3)	C24—C23—C28	119.2 (3)
N4—C4—H4	121.4	C22—C23—C28	123.0 (3)
P2—C4—H4	121.4	C25—C24—C23	121.7 (3)
C6—C5—H5A	109.5	C25—C24—H24	119.2
C6—C5—H5B	109.5	C23—C24—H24	119.2
H5A—C5—H5B	109.5	C26—C25—C24	119.4 (3)
C6—C5—H5C	109.5	C26—C25—H25	120.3
H5A—C5—H5C	109.5	C24—C25—H25	120.3
H5B—C5—H5C	109.5	C25—C26—C27	122.3 (3)
N5—C6—C7	123.2 (3)	C25—C26—H26	118.9
N5—C6—C5	119.6 (3)	C27—C26—H26	118.9
C7—C6—C5	117.2 (3)	C26—C27—C22	117.5 (3)
C8—C7—C6	129.0 (3)	C26—C27—C31	119.4 (3)
C8—C7—H7	115.5	C22—C27—C31	123.2 (3)
C6—C7—H7	115.5	C23—C28—C30	111.5 (3)
N6—C8—C7	122.1 (3)	C23—C28—C29	110.2 (3)
N6—C8—C9	119.2 (3)	C30—C28—C29	110.5 (3)
C7—C8—C9	118.8 (3)	C23—C28—H28	108.2
C8—C9—H9A	109.5	C30—C28—H28	108.2
C8—C9—H9B	109.5	C29—C28—H28	108.2
H9A—C9—H9B	109.5	C28—C29—H29A	109.5
C8—C9—H9C	109.5	C28—C29—H29B	109.5
H9A—C9—H9C	109.5	H29A—C29—H29B	109.5
H9B—C9—H9C	109.5	C28—C29—H29C	109.5
C11—C10—C15	121.9 (3)	H29A—C29—H29C	109.5
C11—C10—N5	119.3 (3)	H29B—C29—H29C	109.5
C15—C10—N5	118.8 (3)	C28—C30—H30A	109.5
C10—C11—C12	116.5 (4)	C28—C30—H30B	109.5
C10—C11—C16	123.5 (3)	H30A—C30—H30B	109.5
C12—C11—C16	120.1 (4)	C28—C30—H30C	109.5
C13—C12—C11	122.2 (5)	H30A—C30—H30C	109.5
C13—C12—H12	118.9	H30B—C30—H30C	109.5
C11—C12—H12	118.9	C32—C31—C27	112.0 (4)
C12—C13—C14	120.7 (4)	C32—C31—C33	110.7 (5)
C12—C13—H13	119.7	C27—C31—C33	111.0 (4)
C14—C13—H13	119.7	C32—C31—H31	107.6
C13—C14—C15	120.5 (5)	C27—C31—H31	107.6
C13—C14—H14	119.8	C33—C31—H31	107.6
C15—C14—H14	119.8	C31—C32—H32A	109.5
C14—C15—C10	118.2 (4)	C31—C32—H32B	109.5
C14—C15—C19	118.9 (4)	H32A—C32—H32B	109.5
C10—C15—C19	122.9 (3)	C31—C32—H32C	109.5
C11—C16—C18	111.3 (4)	H32A—C32—H32C	109.5
C11—C16—C17	112.8 (4)	H32B—C32—H32C	109.5
C18—C16—C17	109.8 (4)	C31—C33—H33A	109.5
C11—C16—H16	107.6	C31—C33—H33B	109.5
C18—C16—H16	107.6	H33A—C33—H33B	109.5
C17—C16—H16	107.6	C31—C33—H33C	109.5
C16—C17—H17A	109.5	H33A—C33—H33C	109.5
C16—C17—H17B	109.5	H33B—C33—H33C	109.5
H17A—C17—H17B	109.5		
